# The Expression of LIGHT Was Increased and the Expression of HVEM and BTLA Were Decreased in the T Cells of Patients with Rheumatoid Arthritis

**DOI:** 10.1371/journal.pone.0155345

**Published:** 2016-05-16

**Authors:** Bin Yang, Zhuochun Huang, Weihua Feng, Wei Wei, Junlong Zhang, Yun Liao, Linhui Li, Xinle Liu, Zhiqiang Wu, Bei Cai, Yangjuan Bai, Lanlan Wang

**Affiliations:** 1 Department of Laboratory Medicine, West China Hospital, Sichuan University, 610041, Chengdu, China; 2 West China Medical School, Sichuan University, 610041, Chengdu, China; Rowan University, UNITED STATES

## Abstract

**Background:**

Currently, the pathogenesis of rheumatoid arthritis (RA) is not clearly understood. The LIGHT/HVEM/BTLA co-signaling pathway may be involved in the pathogenesis of RA, although reports on the expression levels of LIGHT, HVEM and BTLA in T lymphocytes from RA patients are limited.

**Method:**

In this study, we recruited 30 healthy controls and 21 RA patients. Clinical characteristics were collected for RA patients. The levels of LIGHT, HVEM and BTLA expressed on the surface of circulating T cells of RA patients and healthy controls were measured by flow cytometry.

**Result:**

The percentages of CD3+, CD4+ and CD8+ T lymphocytes that expressed BTLA from RA patients were all higher than those of the controls (all *p* < 0.05), while the percentages of CD3+, CD4+ and CD8+ T lymphocytes that expressed HVEM and LIGHT were all lower than those of the controls (all *p* < 0.05). The rheumatoid factor and the percentage of HVEM+CD4+ T lymphocytes showed a statistically significant negative correlation in RA patients (r = -0.453, p = 0.039), as did the swollen joint count and the percentage of BTLA+CD8+ T lymphocytes (r = -0.501, p = 0.021).

**Conclusion:**

Here, we provide the first report on the increased expression of BTLA in T lymphocytes and on the decreased expression of HVEM and LIGHT in RA patients. BTLA, HVEM and LIGHT might be involved in the pathogenesis of RA and have the potential to be new clinically useful characteristics of RA.

## Introduction

T lymphocytes require both co-stimulatory and co-inhibitory signals to be fully activated. Without co-inhibitory or co-stimulatory signal, T lymphocytes become over-activated or anergic [1]. Recently, three proteins expressed on the surface of T lymphocytes were identified: B and T lymphocyte attenuator (BTLA), herpesvirus entry mediator (HVEM) and LIGHT (also known as tumor necrosis factor superfamily 14) [2, 3]. The interactions among these three proteins produce both co-inhibitory and co-stimulatory signals and modulate the activation, proliferation and immune function of T lymphocytes [4].

BTLA is a transmembrane protein with two immunoreceptor tyrosine-based inhibitory motifs in its cytoplasmic tail [2]. Recent studies have revealed that it is expressed on T_H_1 but not T_H_2 lymphocytes, which suggests that BTLA is involved in the T_H_1-mediated immune response [2, 5]. In vitro, btla-/- T lymphocytes are hypo-responsive to suppressive function of regular T lymphocytes because HVEM cannot bind to BTLA. [6, 7]. In vivo, BTLA plays an important role in the induction of the peripheral tolerance of both CD4+ and CD8+ T lymphocytes [8]. In addition, *btla*^*-/-*^ mice have a high level of activated peripheral T lymphocytes and can develop autoimmune hepatitis [9]. These features all indicate the importance of BTLA in autoimmunity. LIGHT is a type-II transmembrane protein that also binds to HVEM [3]. The membrane-bound form of LIGHT produces a co-stimulatory signal through its binding with HVEM and consequently activates T lymphocyte development and proliferation and stimulates the secretion of proinflammatory factors [10, 11]. Moreover, the membrane-bound form of LIGHT is able to disrupt the binding between BTLA and HVEM, which suggests it has a function in the BTLA/HVEM/LIGHT co-signaling pathway [12]. HVEM, as mentioned previously, contributes to both the co-stimulatory and co-inhibitory pathways [13]. This type-1 transmembrane protein functions by binding with its ligands, such as BTLA and LIGHT [14]. *Hvem*^*-/-*^ mice show an increased susceptibility to experimental allergic encephalomyelitis induced by the injection of myelin oligodendrocyte glycoprotein, which is also observed in *btla*^*-/-*^ mice [2, 7]. In general, the BTLA/HVEM/LIGHT co-signaling pathway is highly involved in inflammation and autoimmune reactions.

Rheumatoid arthritis (RA) is a common chronic autoimmune disease that is characterized by persistent synovitis and systemic inflammation [15]. Patients with RA suffer from pain and a decreased capacity for daily activities throughout their lives [16]. Although the pathogenesis of RA is not yet clearly understood, the recent consensus is that the development of RA is attributable to both genetic and environmental factors [16]. As mentioned previously, the LIGHT/HVEM/BTLA co-signaling pathway plays an important role in inflammation and autoimmune reactions. Recent studies have revealed that the expression of LIGHT is increased in RA patients [11], and characteristic patterns of the expression and distribution of both BTLA and HVEM have been observed in the synovium of RA patients [17]. HVEM-Ig treatment which blocked LIGHT/LTα could aggravated development and progression of collagen-induced arthritis in an animal model[18]. Moreover, two single-nucleotide polymorphisms of the BTLA gene (800T and 590C) have been reported to be associated with RA susceptibility [19, 20]. These studies all suggest that the BTLA/HVEM/LIGHT co-signaling pathway is involved in the pathogenesis of RA, even though none of these studies measured the expression levels of BTLA, HVEM or LIGHT in T lymphocytes, which are all vital for the functional performance of the LIGHT/HVEM/BTLA co-signaling pathway [1, 21].

The goal of our study was to determine the differences in the expression levels of BTLA, HVEM and LIGHT in T lymphocytes in RA patients compared to those without RA. The results showed that the expression levels of BTLA and HVEM in T lymphocytes were increased and that of LIGHT was decreased in RA patients. Our findings suggest that the LIGHT/HVEM/BTLA co-signaling pathway plays a role in pathogenesis of RA.

## Materials and Methods

### Participants

This study was approved by the Institutional Review Board of Sichuan University. Twenty-one RA patients and 30 healthy control subjects were recruited from the West China Hospital, Sichuan University between April 2014 and September 2014. All participants provided their written informed consent to participate in this study. All RA patients were diagnosed by rheumatology specialists using the 1987 diagnostic criteria of the American College of Rheumatology [22]. At inclusion, all patients had moderate to severe active disease and were consistent with the criteria of activity: ⑴ ≥ 4 joints were swollen; ⑵ ≥ 6 joints were tender; and ⑶ fit to one of the three criteria: ① morning stiffness lasts more than 45 min; ② erythrocyte sedimentation rate (ESR) ≥ 28 mm/h; ③ C-reactive protein (CRP) > 10 mg/l. All RA patients had been receiving methotrexate (MTX) treatment (10–15 mg per week) for at least one year prior to the study. All healthy controls had no history of RA, ankylosing spondylitis and systemic lupus erythematosus.

### Clinical characteristics and blood samples

Clinical data of RA patients [C-reactive protein (CRP), rheumatoid factor (RF), erythrocyte sedimentation rate (ESR), swollen joint count and tender joint count] were collected at the time of recruitment.

Blood samples were collected from RA patients and from healthy controls when they were recruited for this study. Fasting blood samples (3 ml) were drawn into a BD Vacutainer tubes containing sodium heparin for flow cytometry.

### Flow cytometry of LIGHT, HVEM and BTLA on T lymphocytes

For the analysis of HVEM and BTLA on the surface of T cells, 50 μl of whole blood was incubated with fluorochrome-conjugated anti-human antibodies specific for CD3, CD4, CD8, and with biotin-labeled anti-human antibodies specific for BTLA, isotype control of BTLA, HVEM or isotype control of HVEM, in the dark at 4°C for 30 min. After incubation, streptavidin-PE was added, and the samples were incubated in the dark at 4°C for 30 min. Cells were then hemolyzed and washed with PBS. The antibodies used were PerCP-labeled anti-human CD3, FITC-labeled anti-human CD4, APC-labeled anti-human CD8, biotin-labeled anti-human BTLA, biotin-labeled anti-human HVEM and streptavidin-PE (eBioscience, San Diego, California, USA).

For the analysis of LIGHT expression in T cells, 50 μl of whole blood from each sample was cultured in complete culture medium (RPMI 1640 supplemented with 10% heat-inactivated fetal calf serum) for 4 h in the presence of phorbol myristate acetate (PMA, 10 ng/ml) and ionomycin (1 μg/ml) in incubators set at 37°C in a 5% CO_2_ environment. After cell preparation, fluorochrome-conjugated anti-human antibodies specific for CD3 and CD8, and LIGHT or its isotype control were incubated with the stimulated samples in the dark at 4°C for 30 min. Then, after hemolysis, cells were washed with PBS. CD4-positive cells can become CD4-negative cells after stimulation with PMA due to the endocytosis of the CD4 molecules caused by PMA. Therefore, we used anti-human CD3 and CD8 for surface staining and analyzed CD3+CD8- cells instead of CD3+CD4+ cells. The antibodies used were PerCP-labeled anti-human CD3, APC-labeled anti-human CD8 and PE-labeled anti-human LIGHT (eBioscience, San Diego, California, USA) [23].

Isotype controls were used to enable correct compensation and to confirm antibody specificity (S1 Fig). Stained cells were run through a FACSCanto cytometer (BD Bioscience), and the data were analyzed using FACSDiva software (BD Bioscience). According to the manufacturer’s sheets (BD Bioscience), blocking step wasn’t necessary, so in our FCM analysis, we didn’t apply the block step in surface antigens detection. This method was described in detail in our previous publication [24].

### Statistical analysis

Values are expressed as the mean ± SD or the median with the interquartile range. Data were analyzed using SPSS ver. 19.0 (Chicago, IL). Differences in sex were tested by a χ2 test. Differences in age and the clinical characteristics between RA patients and healthy controls were tested with a two-tailed Mann-Whitney U test. Correlations between the percentages of different T lymphocyte types and the clinical characteristics were tested with the Spearman test. A *p* < 0.05 was considered statistically significant.

## Results

### Participants

Our study recruited 21 RA patients (average age 42 years, range 30 to 60 years, 4 men and 17 women) and 30 healthy control subjects (average age 46 years, range 21 to 60 years, 6 men and 24 women). There were no significant differences in the age or sex of subjects in the RA patient and control groups (Table 1).

**Table 1:** Clinical characteristics of patients (n = 21) and controls (n = 30). CRP: C-reactive protein, RF: rheumatoid factor, ESR: erythrocyte sedimentation rate. Values are expressed as the median with the interquartile range. *p* < 0.05 was considered statistically significant.

Characteristics	Healthy controls		RA patients		*p*
Sex (male: female)	6: 24		4: 17		0.933
Age (years)	42	(39–58)	46.00	(40–52)	0.811
CRP (mg/l)			2.90	(1.50–12.19)	
RF (IU/ml)			124.00	(31.65–358.50)	
ESR (mm/h)			16.00	(10.00–27.50)	
Swollen joint count (0–28, n)			14	(10–17)	
Tender joint count (0–28, n)			11	(6–13)	

### Expression levels of BTLA, HVEM and LIGHT in T lymphocytes

**Fig 1:**
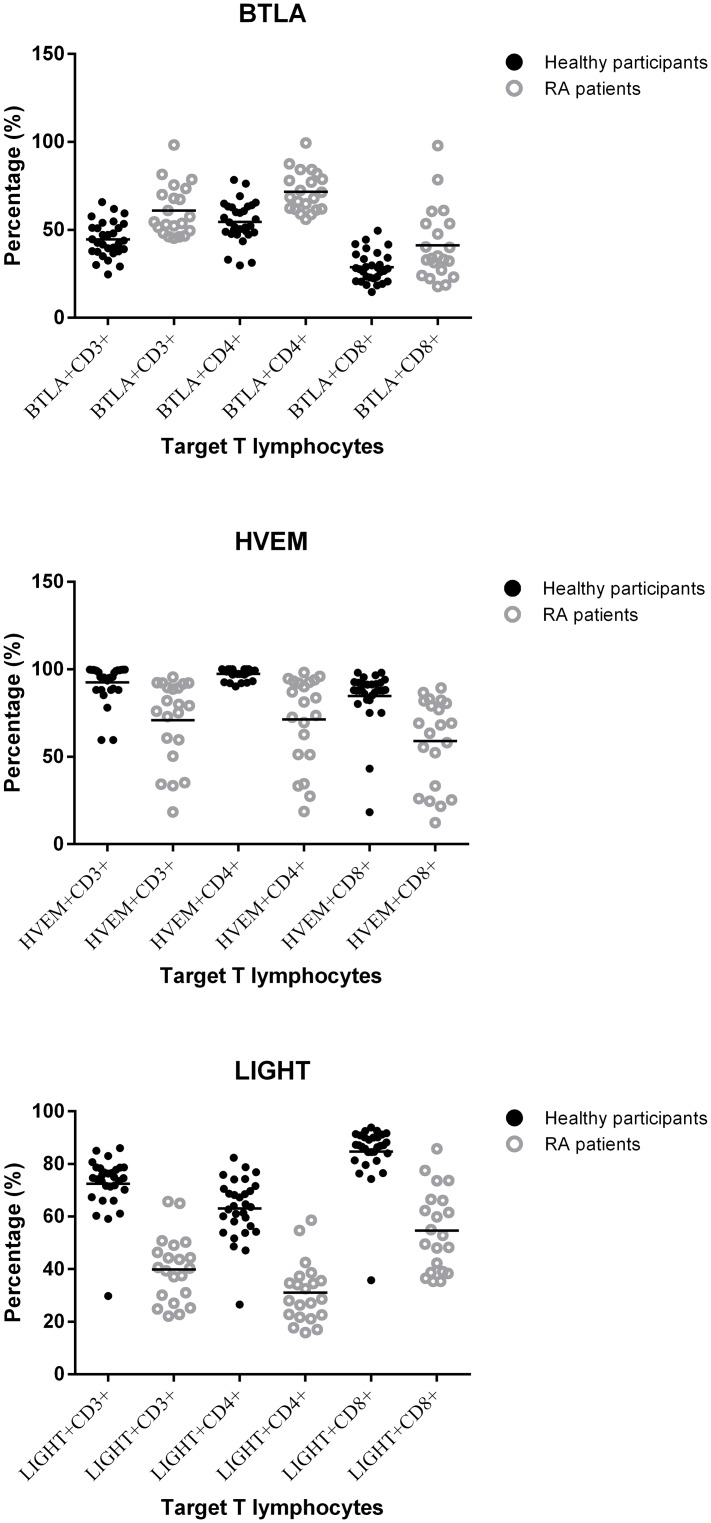
Percentage of BTLA+, HVEM+ or LIGHT+ T lymphocytes. All percentages of T lymphocytes were statistically significantly different between RA patients and healthy controls.

**Fig 2:**
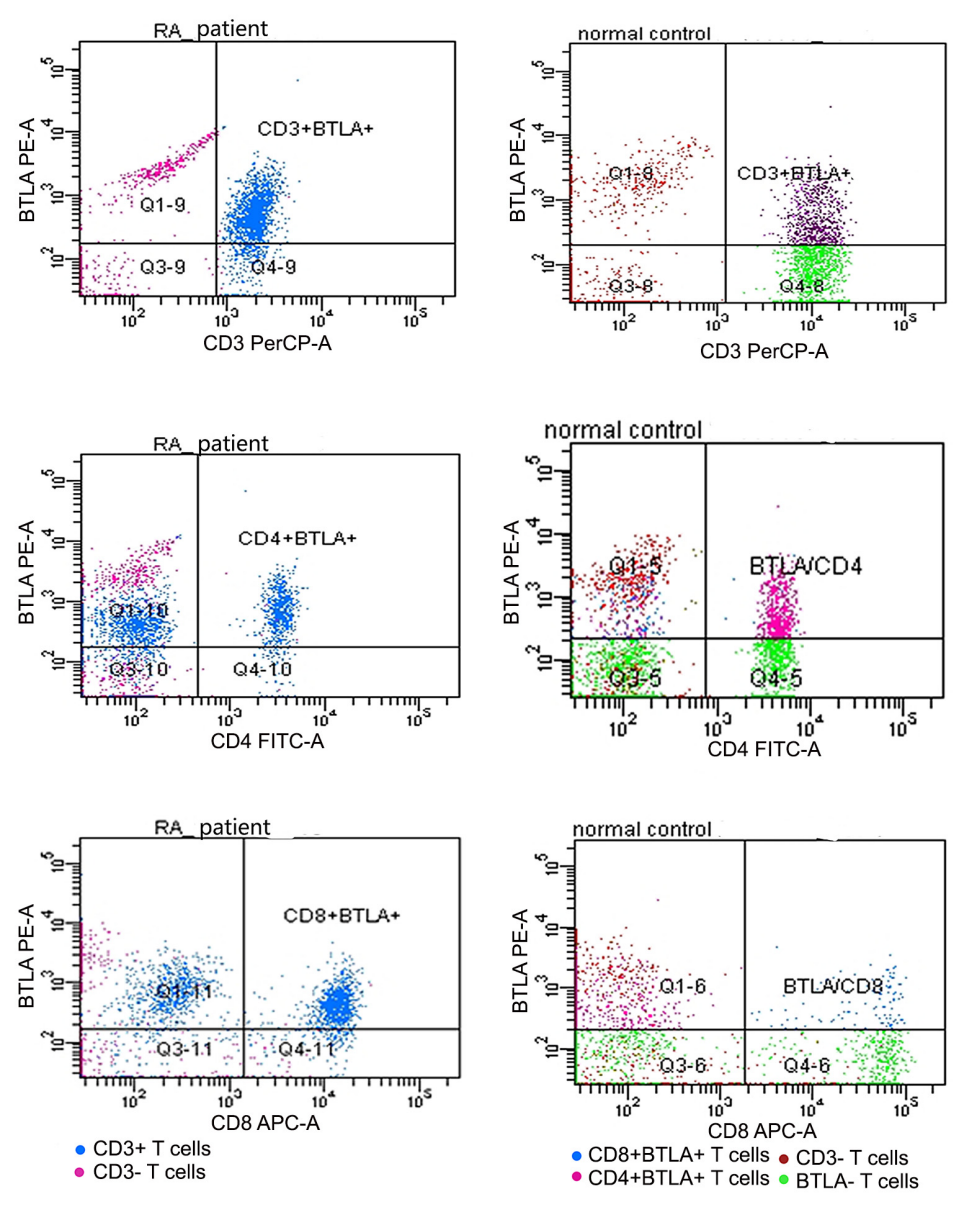
Expression of BTLA on peripheral T cells. T cells of RA patients and healthy controls ran in different times, so the same color might stand for different subtypes of T cells. Dots above to the horizontal line stood for BTLA+ T cells, inferior to the horizontal line stood for BTLA- T cells, right to the vertical line stood for CD3+, CD4+ or CD8+ T cells and left to the vertical line stood for CD3-, CD4- or CD8- T cells.

**Fig 3:**
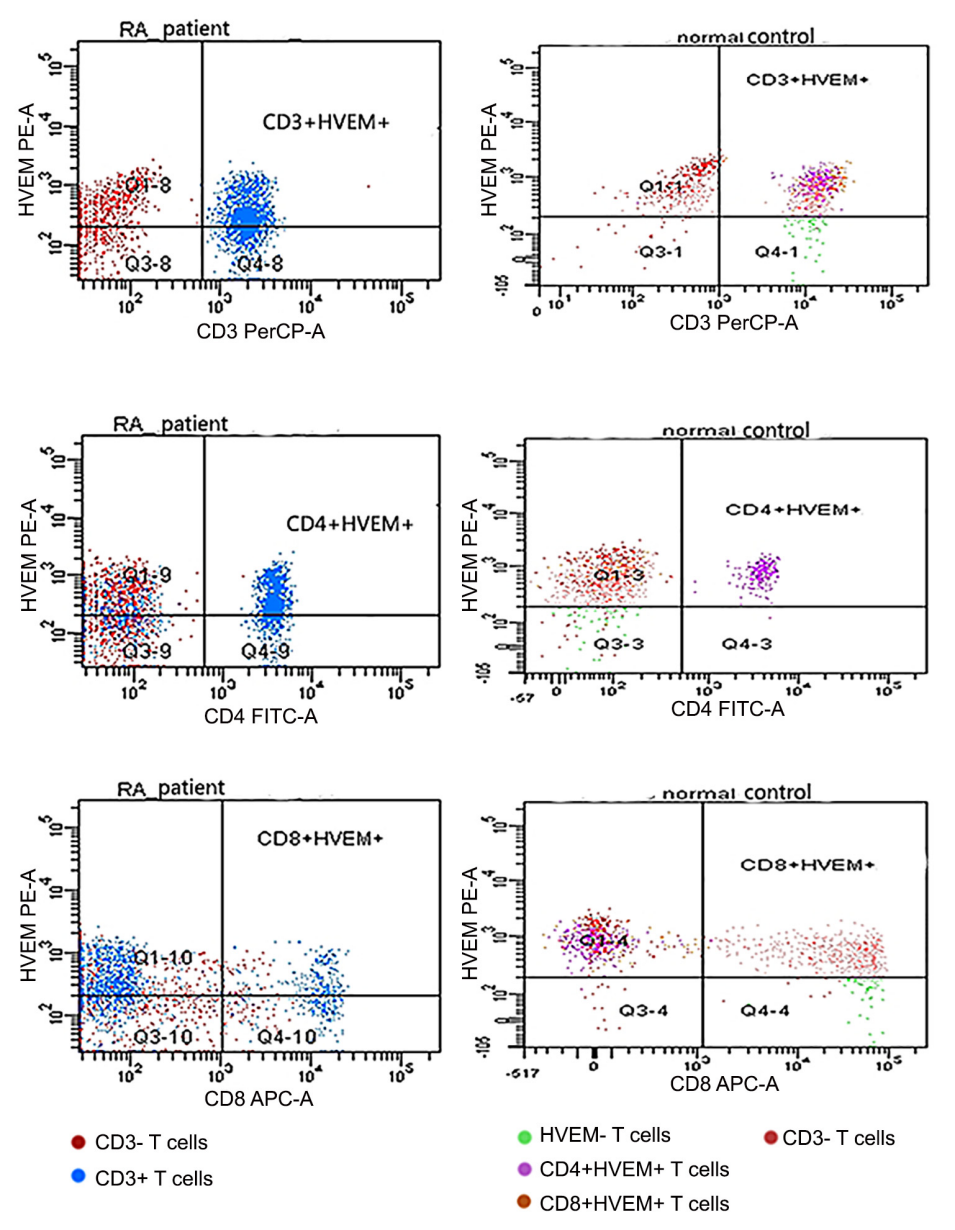
Expression of HVEM on peripheral T cells. T cells of RA patients and healthy controls ran in different times, so the same color might stand for different subtypes of T cells. Dots above to the horizontal line stood for HVEM+ T cells, inferior to the horizontal line stood for HVEM- T cells, right to the vertical line stood for CD3+, CD4+ or CD8+ T cells and left to the vertical line stood for CD3-, CD4- or CD8- T cells.

**Fig 4:**
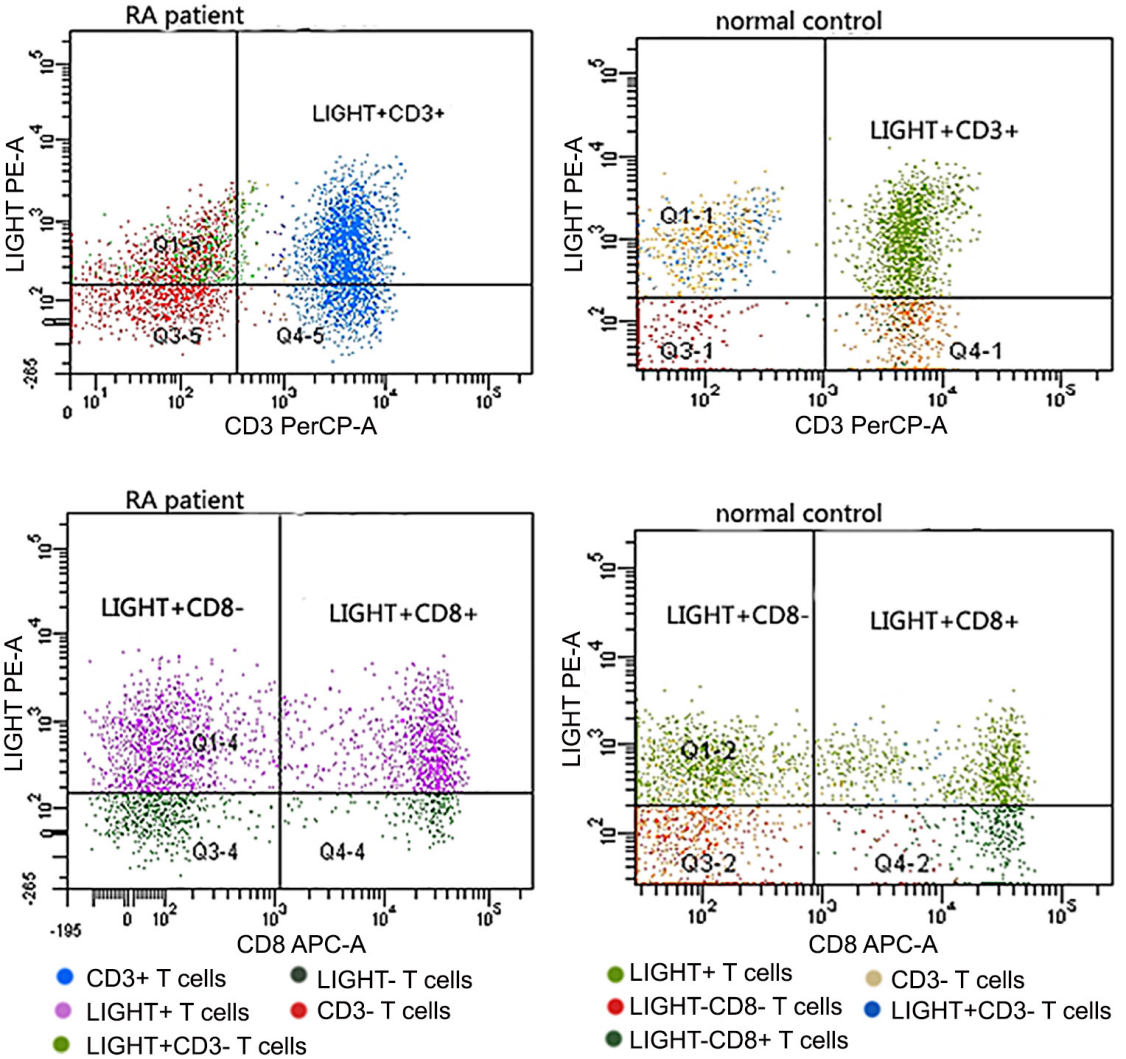
Expression of LIGHT on peripheral T cells. T cells of RA patients and healthy controls ran in different times, so the same color might stand for different subtypes of T cells. Dots above to the horizontal line stood for LIGHT+ T cells, inferior to the horizontal line stood for LIGHT- T cells, right to the vertical line stood for CD3+ or CD8+T cells and left to the vertical line stood for CD3- or CD8- T cells. CD8- T cells stood for CD4+ T cells.

The percentage of CD3+, CD4+ and CD8+ T lymphocytes that expressed BTLA, HVEM and LIGHT were all significantly different between controls and RA patients (all *p* < 0.05) (Table 2, Figs 1, 2, 3 and 4). Expressions of BTLA and HVEM were studied in freshly isolated T cells while expression of LIGHT was studied in activated T cells. The percentages of CD3+, CD4+ and CD8+ T lymphocytes that expressed BTLA were all higher in RA patients than in controls while the percentages of CD3+, CD4+ and CD8+ T lymphocytes that expressed HVEM and LIGHT were all lower in RA patients than those in controls.

**Table 2:** Percentages of BTLA+, HVEM+ or LIGHT+ T lymphocytes. Values are expressed as the mean ± SD with the range. *p* < 0.05 was considered statistically significant.

Characteristics	Healthy participants (n = 30)		RA patients (n = 21)		*p*
Target T lymphocytes					
BTLA+CD3+ (%)	44.6 ± 10.1	(24.7–65.8)	60.9 ± 14.5	(45.3–98.3)	**8.70E-05**
BTLA+CD4+ (%)	54.6 ± 11.7	(29.8–79.4)	71.6 ± 11.5	(56.1–99.4)	**2.10E-05**
BTLA+CD8+ (%)	28.8 ± 8.6	(14.7–49.5)	41.2 ± 20.4	(17.8–97.9)	**0.022**
HVEM+CD3+ (%)	92.5 ± 10.5	(59.6–99.8)	70.9 ± 23.6	(18.4–95.5)	**2.05E-08**
HVEM+CD4+ (%)	97.3 ± 2.9	(90.0–100.0)	71.3 ± 25.5	(19.0–98.0)	**3.55E-08**
HVEM+CD8+ (%)	84.7 ± 16.1	(18.3–98.0)	59.0 ± 25.0	(12.3–89.3)	**6.40E-08**
LIGHT+CD3+ (%)	72.4 ± 10.5	(29.8–86.0)	39.9 ± 12.5	(22.1–65.7)	**3.00E-05**
LIGHT+CD4+ (%)	63.1 ± 11.4	(26.5–82.4)	31.0 ± 11.4	(15.9–58.6)	**2.37E-07**
LIGHT+CD8+ (%)	84.7 ± 10.5	(36.0–94.0)	54.6 ± 15.4	(35.0–86.0)	**5.00E-06**

### Correlations between BTLA, HVEM or LIGHT and clinical characteristics

The RF values and the percentage of HVEM+CD4+ T lymphocytes showed a statically significant negative correlation (r = -0.453, *p* = 0.039), as did the swollen joint count and percentage of BTLA+CD8+ T lymphocytes (r = -0.501, *p* = 0.021) (Table 3). The percentages of other T lymphocytes did not show a statically significant correlation with any clinical characteristics (all *p* > 0.05) (Table 3).

**Table 3:** Correlation between BTLA, HVEM or LIGHT and clinical characteristics. Values are expressed as r (*p*). *p* < 0.05 was considered statistically significant.

Clinical characteristics	CRP	RF	ESR	Swollen joint count	Tender joint count
Target T lymphocytes					
BTLA+CD3+ (%)	-0.201 (0.383)	-0.130 (0.574)	-0.007 (0.975)	-0.366 (0.102)	-0.088 (0.703)
BTLA+CD4+ (%)	-0.205 (0.373)	-0.291 (0.200)	-0.031 (0.893)	-0.388 (0.082)	-0.060 (0.795)
BTLA+CD8+ (%)	-0.232 (0.312)	-0.220 (0.338)	-0.201 (0.383)	**-0.501 (0.021)**	-0.239 (0.297)
HVEM+CD3+ (%)	0.046 (0.844)	-0.369 (0.100)	-0.088 (0.703)	0.109 (0.638)	0.212 (0.357)
HVEM+CD4+ (%)	0.039 (0.867)	**-0.453 (0.039)**	-0.017 (0.492)	0.080 (0.732)	0.167 (0.470)
HVEM+CD8+ (%)	-0.036 (0.878)	-0.272 (0.233)	-0.088 (0.704)	-0.010 (0.964)	0.198 (0.389)
LIGHT+CD3+ (%)	0.186 (0.418)	-0.315 (0.165)	0.076 (0.745)	0.224 (0.330)	0.242 (0.290)
LIGHT+CD4+ (%)	0.305 (0.179)	-0.129 (0.578)	0.072 (0.757)	0.256 (0.262)	0.256 (0.263)
LIGHT+CD8+ (%)	0.114 (0.624)	-0.332 (0.141)	0.230 (0.316)	0.431 (0.051)	0.395 (0.077)

## Discussion

BTLA, HVEM and LIGHT produce co-inhibitory and co-stimulatory signals for the appropriate activation, development and proliferation of T lymphocytes [1, 8, 25]. Binding between BTLA and HVEM produces inhibitory signals and binding between LIGHT and HVEM produces stimulatory signals. Both of those pathways are likely to be involved in inflammation and autoimmune reactions. In the present study, we found that the expression of LIGHT on activated CD3+, CD4+ and CD8+ T lymphocytes in RA patients was decreased compared with that of healthy controls. This was consistent with the result of Edwards et al. [26], who showed that the concentration of the soluble form of LIGHT was increased in the serum of RA patients [26]. LIGHT could change to soluble form from membrane-bound form. Soluble form of LIGHT binds to HVEM and disrupts the binding between the membrane-bound form of LIGHT and HVEM and then inhibits the co-stimulating signal [4, 27, 28]. Kang et al. and Morel et al. observed no LIGHT expression in freshly isolated T cells [29, 30]. Activation with PMA/Ionomycin for 24h led to a small increase in LIGHT expression in the Kang et al. study [29]. Morel at al. observed 40% LIGHT+ T cells after 6 hours stimulation with PMA/Ionomycin [30]. Considering that Antibodies against LIGHT from different providers (Our antibodies against LIGHT were provided by eBioscience, San Diego, California, USA, which was different from Kang et al. and Morel et al.) and activation of T cells both could lead to the high frequency of LIGHT+ T cells, and that T cells of RA patients and healthy controls in the present study were both activated, we thought our result could indicate a difference in expression of LIGHT in T cells between RA patients and healthy controls. These findings suggest that the co-stimulating signal produced by the binding between the membrane-bound form of LIGHT and HVEM is inhibited in RA patients.

We also found a decreased expression of HVEM on CD3+, CD4+ and CD8+ T cells and increased expression of BTLA on CD3+, CD4+ and CD8+ T cells in RA patients, which supports the findings of Shang et al., who showed characteristic patterns of the expression and distribution of BTLA and HVEM in the synovium of RA patients [17]. Kang et al. stated HVEM expression levels in CD3+ lymphocytes from peripheral blood were lower in RA patients than in health controls though the difference was not statistically significant [29]. Because BTLA may bind to HVEM as a co-inhibitory signal and suppress inflammation and autoimmune reactions, an increase in its expression in T lymphocytes might be considered to be self-regulation or the effect of MTX therapy. BTLA expression in the present study is low in healthy controls compared to study of Otsuki et al. (44.6% BTLA+ T cells *vs*. 90% BTLA+ T cells) [30], while in another study, expression level of in T cells was similar to our observation (near 50% BTLA+ T cells) [31]. In addition, our provider of antibodies against HVEM (eBioscience, San Diego, California, USA) and our samples—which were all from Han Chinese population—were both different from the study of Otsuki et al. For those reasons, a difference in the result could be possible.

To determine whether BTLA, HVEM and LIGHT could be used as new clinical markers of RA, we measured the correlation between the percentage of BTLA+, HVEM+ or LIGHT+ T lymphocytes and the clinical characteristics for RA patients (CRP, RF, ESR, swollen joint count and tender joint count). Only two pairs of characteristics (HVEM+CD4+ T lymphocytes and RF, BTLA+CD8+ T lymphocytes and ESR) showed statistically significant negative correlations. Based on these results and the fact that the RA patients in our study had already received long-term MTX therapy, BTLA, HVEM and LIGHT might be potentially useful clinical characteristics of RA.

Our study had several limitations. It included a small number of participants (21 RA patients and 30 healthy controls), and the RA patients were not treatment-naïve. These limitations were due to the difficulty in recruiting volunteers and could lead to inaccurate results. In addition, we only evaluated the percentage of T lymphocytes that expressed BTLA, HVEM or LIGHT in the serum, which are not as representative of the pathogenesis of RA as the levels in the synovial fluid of arthritic joints. A future study that uses synovial fluid from arthritic joints is needed to complete our study.

In conclusion, here we provide the first report that the expression of BTLA in T lymphocytes is increased and the expression of HVEM and LIGHT are decreased in RA patients. BTLA, HVEM and LIGHT might be involved in the pathogenesis of RA and have the potential to serve as new clinical characteristics of RA. A future study with more participants and treatment-naïve RA patients that also measures the expression of BTLA, HVEM and LIGHT in arthritic synovial fluid is needed.

## Supporting Information

S1 Fig Isotype control histogram for FCM analysis. Blank above to the horizontal line stood for BTLA+, HVEM+ or LIGHT+ T cells. Dots inferior to the horizontal line stood for BTLA-, HVEM- or LIGHT- T cells, right to the vertical line stood for CD3+, CD4+ or CD8+ T cells and left to the vertical line stood for CD3-, CD4- or CD8- T cells.
(TIF)

S1 Table Data of RA patients and healthy controls. (XLSX)

S1 File STROBE_checklist_v4_combined_PlosMedicine. (DOCX)
