# Correction: The Expression of LIGHT Was Increased and the Expression of HVEM and BTLA Were Decreased in the T Cells of Patients with Rheumatoid Arthritis

**DOI:** 10.1371/journal.pone.0173531

**Published:** 2017-03-02

**Authors:** Bin Yang, Zhuochun Huang, Weihua Feng, Wei Wei, Junlong Zhang, Yun Liao, Linhui Li, Xinle Liu, Zhiqiang Wu, Bei Cai, Yangjuan Bai, Lanlan Wang

In the title of this article, the words “LIGHT” and “BTLA” are switched. The correct title is: The Expression of BTLA Was Increased and the Expression of HVEM and LIGHT Were Decreased in the T Cells of Patients with Rheumatoid Arthritis. The correct citation is: Yang B, Huang Z, Feng W, Wei W, Zhang J, Liao Y, et al. (2016) The Expression of BTLA Was Increased and the Expression of HVEM and LIGHT Were Decreased in the T Cells of Patients with Rheumatoid Arthritis. PLoS ONE 11(5): e0155345. doi:10.1371/journal.pone.0155345.
