# Supplementary figures and images for: The Expression of LIGHT Was Increased and the Expression of HVEM and BTLA Were Decreased in the T Cells of Patients with Rheumatoid Arthritis
Source: PLoS One. 2016 May 16;11(5):e0155345. doi: 10.1371/journal.pone.0155345 (PMC4868345; doi:10.1371/journal.pone.0155345)

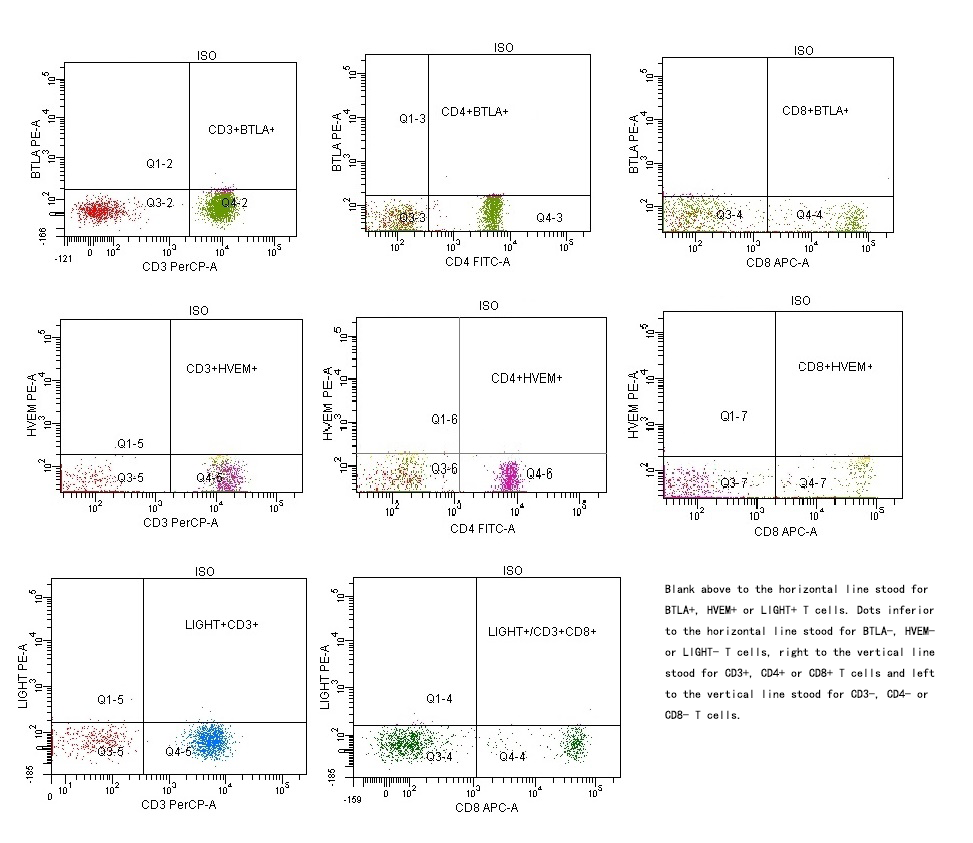

Supplement: S1 Fig — Blank above to the horizontal line stood for BTLA+, HVEM+ or LIGHT+ T cells. Dots inferior to the horizontal line stood for BTLA-, HVEM- or LIGHT- T cells, right to the vertical line stood for CD3+, CD4+ or CD8+ T cells and left to the vertical line stood for CD3-, CD4- or CD8- T cells. (TIF) [file pone.0155345.s001.tif]
