# Supplementary material for: The Expression of LIGHT Was Increased and the Expression of HVEM and BTLA Were Decreased in the T Cells of Patients with Rheumatoid Arthritis
Source: PLoS One. 2016 May 16;11(5):e0155345. doi: 10.1371/journal.pone.0155345 (PMC4868345; doi:10.1371/journal.pone.0155345)
Supplement: S1 File — (DOCX) [file pone.0155345.s003.docx]

STROBE Statement—checklist of items that should be included in reports of observational studies

|  | Item No. | Recommendation | Page  No. | Relevant text from manuscript |
| --- | --- | --- | --- | --- |
| **Title and abstract** | 1 | (*a*) Indicate the study’s design with a commonly used term in the title or the abstract | 2 | Case-control study |
|  |  | (*b*) Provide in the abstract an informative and balanced summary of what was done and what was found | 1 | The expression of LIGHT was increased and the expressions of HVEM and BTLA were decreased on T cells of patients with rheumatoid arthritis |
| Introduction | | | |  |
| Background/rationale | 2 | Explain the scientific background and rationale for the investigation being reported | 2 | Pathogenesis of rheumatoid arthritis (RA) is not understood clearly recently. LIGHT/HVEM/BTLA co-signaling pathway may be involved in pathogenesis of RA, while report about expression levels of LIGHT, HVEM and BTLA on T lymphocytes of RA patients is lacked.. |
| Objectives | 3 | State specific objectives, including any prespecified hypotheses | 5 | Our study aimed to reveal the differences between expression levels of BTLA, HVEM and LIGHT on T lymphocytes in RA patients and that of normal people. |
| Methods | | | |  |
| Study design | 4 | Present key elements of study design early in the paper | 6 | Participants with RA, healthy control, flow cytometry |
| Setting | 5 | Describe the setting, locations, and relevant dates, including periods of recruitment, exposure, follow-up, and data collection | 6 | Twenty-one RA patients and 30 healthy controls had been recruited in West China Hospital, Sichuan University since April 2014 to September 2014. All RA patients were diagnosed by rheumatology specialists using 1987 diagnostic criteria of the American College of Rheumatology. All patients have received methotrexate (MTX) treatment for at least one year (10 – 15 mg per week). |
| Participants | 6 | (*a*) *Cohort study*—Give the eligibility criteria, and the sources and methods of selection of participants. Describe methods of follow-up  *Case-control study*—Give the eligibility criteria, and the sources and methods of case ascertainment and control selection. Give the rationale for the choice of cases and controls  *Cross-sectional study*—Give the eligibility criteria, and the sources and methods of selection of participants | 6 | All RA patients were all fulfilling the 1987 diagnostic criteria of the American College of Rheumatology. |
|  |  | (*b*) *Cohort study*—For matched studies, give matching criteria and number of exposed and unexposed  *Case-control study*—For matched studies, give matching criteria and the number of controls per case |  | N/A |
| Variables | 7 | Clearly define all outcomes, exposures, predictors, potential confounders, and effect modifiers. Give diagnostic criteria, if applicable | 6 | N/A |
| Data sources/ measurement | 8* | For each variable of interest, give sources of data and details of methods of assessment (measurement). Describe comparability of assessment methods if there is more than one group | 6, 7 | For analysis of HVEM and BTLA on T cells, 50μl whole blood was incubated with fluorochrome-conjugated anti-human antibodies specific for CD3, CD4, CD8 and biotin-labeled anti-human antibodies specific for BTLA or HVEM in dark at 4°C for 30 min. After that, streptavidin-PE was added for another more incubation in dark at 4°C for 30min. And then after hemolysis cells were washed with PBS. The antibodies used were Percp-labled anti-human CD3, FITC-labeled anti-human CD4, APC-labeled anti-human CD8, biotin-labeled anti-human BTLA, biotin-labeled anti-human HVEM and streptavidin-PE (eBioscience, San Diego, California, USA).  For analysis of LIGHT on T cells, 50 μl whole blood of every sample was cultured in complete culture medium (RPMI 1640 supplemented with 10 % heat-inactivated fetal calf serum) for 4 h, in the presence of phorbol myristate acetate (PMA, 10 ng/ml) plus ionomycin (1 μg/ml). The incubators were set at 37 °C under a 5% CO2 environment. After cell preparation, fluorochrome-conjugated anti-human antibodies specific for CD3, CD8 and LIGHT were incubated with the stimulated samples in dark at 4°C for 30 min and then after hemolysis cells were washed with PBS (Because CD4 positive cells would turn into CD4 negative cells after stimulation with PMA due to the endocytosis of CD4 molecules caused by PMA, so we used anti-human CD3 and CD8 for the surface staining and analyzed CD3+CD8- cells instead of CD3+CD4+ cells) [20]. The antibodies used were Percp-labeled anti-human CD3, APC-labeled anti-human CD8 and PE-labeled anti-human LIGHT (eBioscience, San Diego, California, USA).  Isotype controls were given to enable correct compensation and confirm antibody specificity. Stained cells were run on a FACSCanto cytometer (BD Bioscience), and the data were analysed using FACSDiva software (BD Bioscience). |
| Bias | 9 | Describe any efforts to address potential sources of bias | 13 | Our study had several insufficient, as few participants (21 RA patients and 30 healthy controls) and that RA patients were not treatment-naïve, which were limited by the difficulty to recruit volunteers and it could lead to an inaccurate result. And we just detected percentages of T lymphocytes which expressed BTLA, HVEM or LITHR in serum, which were not as representative for pathogenesis of RA as those in synovial fluid of Arthritic joints. |
| Study size | 10 | Explain how the study size was arrived at |  | N/A |

Continued on next page

| Quantitative variables | 11 | Explain how quantitative variables were handled in the analyses. If applicable, describe which groupings were chosen and why | 6, 7 | For analysis of HVEM and BTLA on T cells, 50μl whole blood was incubated with fluorochrome-conjugated anti-human antibodies specific for CD3, CD4, CD8 and biotin-labeled anti-human antibodies specific for BTLA or HVEM in dark at 4°C for 30 min. After that, streptavidin-PE was added for another more incubation in dark at 4°C for 30min. And then after hemolysis cells were washed with PBS. The antibodies used were Percp-labled anti-human CD3, FITC-labeled anti-human CD4, APC-labeled anti-human CD8, biotin-labeled anti-human BTLA, biotin-labeled anti-human HVEM and streptavidin-PE (eBioscience, San Diego, California, USA).  For analysis of LIGHT on T cells, 50 μl whole blood of every sample was cultured in complete culture medium (RPMI 1640 supplemented with 10 % heat-inactivated fetal calf serum) for 4 h, in the presence of phorbol myristate acetate (PMA, 10 ng/ml) plus ionomycin (1 μg/ml). The incubators were set at 37 °C under a 5% CO2 environment. After cell preparation, fluorochrome-conjugated anti-human antibodies specific for CD3, CD8 and LIGHT were incubated with the stimulated samples in dark at 4°C for 30 min and then after hemolysis cells were washed with PBS (Because CD4 positive cells would turn into CD4 negative cells after stimulation with PMA due to the endocytosis of CD4 molecules caused by PMA, so we used anti-human CD3 and CD8 for the surface staining and analyzed CD3+CD8- cells instead of CD3+CD4+ cells) [20]. The antibodies used were Percp-labeled anti-human CD3, APC-labeled anti-human CD8 and PE-labeled anti-human LIGHT (eBioscience, San Diego, California, USA).  Isotype controls were given to enable correct compensation and confirm antibody specificity. Stained cells were run on a FACSCanto cytometer (BD Bioscience), and the data were analysed using FACSDiva software (BD Bioscience). |
| --- | --- | --- | --- | --- |
| Statistical methods | 12 | (*a*) Describe all statistical methods, including those used to control for confounding | 8 | Values were expressed as mean ± SD. Data were analyzed using SPSS 19.0 software (Chicago, IL). Differences of gender was tested by χ2 test. Differences of clinical data between pre-therapy period and week 12 of one group were tested by Wilcoxon test. Differences of clinical data between healthy participants and patients in two groups or between patients in two groups in the same time were assessed using Mann-Whitney U test. A probable value of p < 0.05 was considered to be statistically significant. |
|  |  | (*b*) Describe any methods used to examine subgroups and interactions |  | N/A |
|  |  | (*c*) Explain how missing data were addressed |  | N/A |
|  |  | (*d*) *Cohort study*—If applicable, explain how loss to follow-up was addressed  *Case-control study*—If applicable, explain how matching of cases and controls was addressed  *Cross-sectional study*—If applicable, describe analytical methods taking account of sampling strategy |  | N/A |
|  |  | (*e*) Describe any sensitivity analyses |  | N/A |
| Results | | | | |
| Participants | 13* | (a) Report numbers of individuals at each stage of study—eg numbers potentially eligible, examined for eligibility, confirmed eligible, included in the study, completing follow-up, and analysed | 9 | No participant was lost to follow-up. |
|  |  | (b) Give reasons for non-participation at each stage |  | N/A |
|  |  | (c) Consider use of a flow diagram |  | N/A |
| Descriptive data | 14* | (a) Give characteristics of study participants (eg demographic, clinical, social) and information on exposures and potential confounders | 9 | Table 1 |
|  |  | (b) Indicate number of participants with missing data for each variable of interest | 9 | There was no participant with missing data in our study |
|  |  | (c) *Cohort study*—Summarise follow-up time (eg, average and total amount) |  | N/A |
| Outcome data | 15* | *Cohort study*—Report numbers of outcome events or summary measures over time |  | N/A |
|  |  | *Case-control study—*Report numbers in each exposure category, or summary measures of exposure | 9 | Our study recruited 21 RA patients (average age 42 years, range 30 to 60 years) including 4 males and 17 females and 30 healthy controls (average age 46 years, range 21 to 60 years) including 6 males and 24 females. |
|  |  | *Cross-sectional study—*Report numbers of outcome events or summary measures |  | N/A |
| Main results | 16 | (*a*) Give unadjusted estimates and, if applicable, confounder-adjusted estimates and their precision (eg, 95% confidence interval). Make clear which confounders were adjusted for and why they were included | 9, 10 | Table 2 and 3 |
|  |  | (*b*) Report category boundaries when continuous variables were categorized |  | N/A |
|  |  | (*c*) If relevant, consider translating estimates of relative risk into absolute risk for a meaningful time period |  | N/A |

Continued on next page

| Other analyses | 17 | Report other analyses done—eg analyses of subgroups and interactions, and sensitivity analyses |  | N/A |
| --- | --- | --- | --- | --- |
| Discussion | | | | |
| Key results | 18 | Summarise key results with reference to study objectives | 13 | We first reported expression of BTLA on T lymphocytes increased and expressions of HVEM and LIGHT decreased in RA patients. |
| Limitations | 19 | Discuss limitations of the study, taking into account sources of potential bias or imprecision. Discuss both direction and magnitude of any potential bias | 13 | Our study had several insufficient, as few participants (21 RA patients and 30 healthy controls) and that RA patients were not treatment-naïve, which were limited by the difficulty to recruit volunteers and it could lead to an inaccurate result. And we just detected percentages of T lymphocytes which expressed BTLA, HVEM or LITHR in serum, which were not as representative for pathogenesis of RA as those in synovial fluid of Arthritic joints. |
| Interpretation | 20 | Give a cautious overall interpretation of results considering objectives, limitations, multiplicity of analyses, results from similar studies, and other relevant evidence | 13 | BTLA, HVEM and LIGHT might be involved in pathogenesis of RA and had potential to be new clinical characteristics of RA. |
| Generalisability | 21 | Discuss the generalisability (external validity) of the study results | 13 | We just detected percentages of T lymphocytes which expressed BTLA, HVEM or LITHR in serum, which were not as representative for pathogenesis of RA as those in synovial fluid of Arthritic joints. |
| Other information | |  | | |
| Funding | 22 | Give the source of funding and the role of the funders for the present study and, if applicable, for the original study on which the present article is based | 14 | This research was sponsored by the National Natural Science Foundation of China (Nos. 81301496 and 81202354). |

*Give information separately for cases and controls in case-control studies and, if applicable, for exposed and unexposed groups in cohort and cross-sectional studies.

**Note:** An Explanation and Elaboration article discusses each checklist item and gives methodological background and published examples of transparent reporting. The STROBE checklist is best used in conjunction with this article (freely available on the Web sites of PLoS Medicine at http://www.plosmedicine.org/, Annals of Internal Medicine at http://www.annals.org/, and Epidemiology at http://www.epidem.com/). Information on the STROBE Initiative is available at www.strobe-statement.org.
